# New Appliances and Things Medical

**Published:** 1898-07-02

**Authors:** 


### NEW APPLIANCES AND THINGS MEDICAL.

**[We shall be glad to receive, at our Office, 28 & 29, Southampton Street, Strand, London, W.C., from the manufacturers, specimens of all new preparations and appliances which may be brought out from time to time.]**

### VALTINE MEAT GLOBULES.

(The Valentine Extract Co., Limited, St. George's House, Eastcheap, London, E.C.)

These globules contain an extract of meat prepared by the well-known Valentine Co. They are supplied in several sizes, the larger ones for making beef tea, the smaller for swallowing. By this method a larger amount of natural moisture can be retained than has hitherto been found possible or convenient. A special feature in these globules is the addition with each tin of the so-called Valtine flavour pelloids, which are small tabloids containing flavouring condiments. They are supplied in varying strengths, and can be added according to taste. This method of globules for swallowing, with or without the combined flavouring, opens up a new field for convenient and scientific invalid feeding.

### ORTHOFORM.

(Meister Lucius and Brüning, 46, St. Mary Axe, London, E.C.)

Orthoform is a new antiseptic and anæsthetic powder for the treatment and relief of painful wounds—ulcers, burns, excoriations, and allied conditions. It is said to be absolutely non-poisonous, and, from experiments which have been published concerning its practical uses, for ordinary purposes this statement must be regarded as proved. Chemically, this new body is para-amido-meta oxy-benzoic acid, and closely allied to cocaine; but, as compared with the latter, orthoform dissolves very slowly in water, although its anæsthetic properties develop quickly and are longer maintained. To its slow solubility must be attributed its chief advantage over allied bodies. By its use a condition of anæsthesia can be maintained for hours or days—a very valuable quality when treating such conditions as burns or ulcers, which deprive the patient of rest and sleep owing to the persistent pain attending these lesions. In addition to its anæsthetic powers, orthoform appears to be a mild antiseptic with desiccative properties. For surface wounds orthoform in the form of powder seems best adapted. For internal use, as in gastric ulcers, the soluble hydro-chloride is stated to be more applicable; but for the latter condition, as also in ophthalmic surgery, orthoform appears to possess no advantage over cocaine, or, if it has, the proofs are still wanting. Its anæsthetic properties, when the soluble hydro-chloride is used, are probably no less evanescent than cocaine, and certainly not nearly so powerful. As an anæsthetic, its use will probably be limited to such cases as burns and ulcers with exposed nerve endings, when its non-poisonous properties and powers of maintaining anæsthesia for long periods of time should prove very valuable.

### EXTRACT RADIAR PERNIONIS.

(Arthur and Co. 69, Berners Street, London.)

This extract has been carefully prepared by Messrs. Arthur and Co. It is a very strong alcoholic extract of a tuber. Its properties are soothing and astringent. It has been specially recommended for itching, bruises, and chilblains, and for these everyday troubles, which often lead to a visit to a quack, it appears to us to be a most useful application—improvement, if not cure, being effected in the cases where we have tried this new extract.

